# Mortality at the pediatric emergency unit of the Mohammed VI teaching hospital of Marrakech

**DOI:** 10.1186/s12873-020-00352-9

**Published:** 2020-07-23

**Authors:** W. Lahmini, M. Bourrous

**Affiliations:** Department of Paediatric Emergency, UHC Mohamed VI, Cadi Ayyad University, PO Box: 7010, Sidi Abbad Street, 40000 Marrakech, Morocco

**Keywords:** Pediatric mortality, Pediatric emergencies, Etiologies, Prevention

## Abstract

**Background:**

The death of a child at the emergency ward is one of the most difficult problems that the clinicians of these wards have to deal with. In our country the published data concerning the causes and the factors related to pediatric mortality especially in the pediatric emergency wards is very rare. This study aimed to study the epidemiology of the pediatric mortality in the pediatric emergency department (PED), to determine its rate and identify its most frequent causes.

**Methods:**

It is a retrospective and descriptive study, over five years (1st January 2012 and 31st December 2016) including all children aged from 0 to 15 years old who died at the PED in the Mohamed VI Hospital in Marrakech.

**Results:**

During the period of the study a total of 172.691 patients presented to the PED, among which 628 died (pediatric mortality rate: 3.63%). The masculine gender was predominant (*n* = 383) with a gender ratio of 1.59. Two-thirds of the patients died in the first 24 h (*n* = 421). The median of time from admission to death was around 12 h. Majority of the deceased children (*n* = 471, 75%) were from a low socioeconomic status. The most frequent cause of admissions for deceased patients in the PED was respiratory distress (*n* = 296, 47%) followed by neurological disorders (*n* = 70, 11%). Neonatal mortality (≤ 1 month of age) was predominant (*n* = 472, 75.1%), followed by postnatal mortality (1 month to 1 year old) (*n* = 73, 11.6%). The most frequent causes of pediatric mortality, whatever the age range, were dominated by neonatal pathologies (*n* = 391, 62.3%), followed by infecious causes bronchopulmonary infections included (*n* = 49, 7.7%), birth deformities (*n* = 46, 7.3%) while traumas were merely at 0.9% (*n* = 6). The most frequent causes of neonatal mortality were neonatal infections (*n* = 152, 32.2%) and prematurity (*n* = 115, 24.4%).

**Conclusion:**

Our data once again underline the crucial importance of prevention. This requires correct follow-up of the pregnancies, an adequate assistance of births, and perfecting healthcare provision to newborns in order to attain proper assistance.

## Background

The death of a child at the emergency ward is one of the most difficult problems that the clinicians of these wards have to deal with [1]. At the international level, important progress has been made in reducing infantile mortality mostly in children under five years old. Indeed, the mortality rate of children under five years old has seen a global decline between 2000 and 2016 going from 69,4 to 38,4 for 1000 live births. In Morocco, despite its decrease from 80 to 27 for 1000 live births from 1990 to 2016, the under five years old mortality is still a public health priority [2]. Pediatric emergency department only exist in University Hospital Centers (tertiary level). In the majority of provinces of our country, children are managed by general practitioners overwhelmed by adult emergencies and not trained in the management of pediatric emergencies. In addition, these emergency structures are not adapted to receive and to treat children in vital distress. This imbalance leads to a tremendous influx at the pediatric emergency departments of the tertiary level, resulting in serious overcrowding that could influence the quality of emergency care for critically ill children.

On the other hand, in our country the published data concerning the causes and the factors related to pediatric mortality especially in the pediatric emergency wards is very rare. Therefore, we aim through this study, to determine the rate of the pediatric mortality in the pediatric emergency department (PED), to study its epidemiology and to identify its most frequent causes.

## Methods

It is a retrospective and descriptive study, over five years, of files that belonged to the deceased children of the PED in the Mohamed VI Hospital in Marrakech, reviewed with a pre established document. The study’s population consisted of children aged from 0 to 15 years old who died at the PED between 1st January 2012 and 31st December 2016. We divided the deaths into 4 groups on the basis of age at death:

-Neonatal mortality: death between birth and the 28th day after birth.
-Postnatal mortality: death between the 29th day and the 1st year of life.
-Toddlers and preschoolers mortality: death between the 1st and the 5th year of life.
-The mortality of children aged between 5 and 15 years old.

Our ward was open in July 2008 and serves not only the entire region of Marrakech but also all cities and provinces of the south of Morocco. It includes a reception and triage unit, with a care space for patients with trauma, a resuscitation unit (2 beds), and 12 beds for short-term hospital for patients monitoring. Its activity didn’t stop to increase going from 21.289 in 2008 to 70.176 admissions in 2018.

Only children aged between 0 and 15 years old and who died in the PED were included in this study. Children that were dead on arrival at the emergency unit were excluded from this study. We mainly reviewed the socio-demographic and clinical characteristics of the deceased patients; the causes of deaths, the date and the time of death. The rural or urban geographic origin has been checked in electronic health record based on home address delivered by the families and the geography of the area of Marrakech. The medical assistance system (MAS) is one of the basic components of the social development policy in Morocco to improve and generalize the health coverage system. This system is dedicated for low-income individuals and families to guarantee free healthcare by delivering their membership card to the hospial. So, the socioeconomic status was checked by using this parameter marked automatically in patient’s electronic chart. Other additional informations were obtained from the paper file. The disease category causes have been defined and classified based on the patient’s file and the International Classification of Diseases (ICD) taking into account the different pediatric age categories. The statistical data were analyzed by the SPSS software. Conditions of confidentiality and anonymous status were thoroughly respected.

## Results

During the period of the study (2011–2016) a total of 172.691 patients presented, among which 628 died in the PED, leading to a pediatric mortality rate calculated at 3.63 per 1000 over 5 years. The masculine at any age was predominant (*n* = 383, 61%) with a gender ratio of 1.59. Neonatal mortality was predominant (*n* = 472, 75.1%) followed by postnatal mortality (*n* = 73, 11.6%). Toddlers and preschoolers mortality was at 7.3% (*n* = 46) and the one for children aged between 5 and 15 years old was at 5.8% (*n* = 37). The most frequent cause of admissions for deceased patients was respiratory distress (*n* = 296, 47%) followed by neurological disorders (*n* = 70, 11%), prematurity (*n* = 44, 7%) and lastly fever (*n* = 26, 4%).

The most frequent causes of pediatric mortality, whatever the age range, were dominated by neonatal pathologies (*n* = 391, 62.3%), followed by infecious causes bronchopulmonary infections included (*n* = 48, 7.7%) and last but not least birth deformities (*n* = 46, 7.3%) while traumas were merely at 0.9% (*n* = 6) (Table 1). Majority of the deceased children (*n* = 471, 75%) were from a low socioeconomic status. Their geographic origin was mostly rural (*n* = 321, 51%). More than two thirds (*n* = 421, 67%) consulted directly in the PED, while (*n* = 189, 30%) were referred from an external health institution. Most of the deaths (*n* = 251, 40%) occurred in the winter season. Majority of the deaths (*n* = 428, 68%) were recorded between the time range of 4 pm to 8 pm. The deceased children at the PED spent a median of time around 12 h. Two-thirds of the patients (*n* = 421, 67%) died in the first 24 h, among which (*n* = 63, 10%) died in the first hour after their admission (Table 2). The most frequent causes of neonatal mortality were: neonatal infections (*n* = 152, 32.2%), prematurity (*n* = 115, 24.4%) and perinatal asphyxia (*n* = 71, 15%) (Table 3). The causes of the postnatal mortality were dominated by pneumonia (*n* = 19, 27.1%) followed by birth defects (*n* = 13, 18.5%). The causes of the Toddlers and preschoolers mortality were dominated equally by pneumonia, meningitis, birth defects and neoplasia with a rate of 11.1% (*n* = 5) each. The mortality causes of children aged between 5 and 15 years old were mainly neoplasic pathologies (*n* = 9, 23.6%) (Table 4). No autopsy was performed during the study period.

**Table 1:** Main causes of pediatric mortality for all ages

Causes of death	Number of deaths	Percentage
Neonatal infections	152	24,2%
Prematurity	115	18,3%
Perinatal asphyxia	71	11,3%
Neonatal respiratory distress	57	9%
Infectious causes	24	3,8%
Pulmonary infections	25	3,9%
Malformations	46	7,3%
Heart Diseases	13	2%
Neoplasia	16	2,5%
Uncertain	96	15,2%
Poisoning	9	1,4%
Traumatic causes	6	0,9%
Various	18	0,4%

**Table 2:** Demographic characteristics and chronological distribution of deaths

VARIABLES	NUMBER	PERCENTAGE
**Gender** (ratio: 1,59)		
Girl	245	39%
Boy	383	61%
**Age (0 days to 15 years)**		
≤ 1 month	472	75,1%
1 month^− 1^ year	73	11,6%
1–5 years	46	7,3%
5–15 years	37	5,8%
**Socio-economic level**		
Low	471	75%
Average	138	22%
High	19	03%
**Geographic origin**		
Rural	320	51%
Suburban	94	15%
Urban	214	34%
**Time of death after admission (median**: **12 h)**		
≤ 01 h	63	10%
01–12 h	138	22%
12–24 h	220	35%
24–48 h	138	22%
≥ 48 h	69	11%
**Reference mode**		
Self-reference	433	69%
Referral from another health center	195	31%

**Table 3:** Etiologies of neonatal mortality

Causes of death	Number of deaths	Percentage
Neonatal infection	152	32,2%
Prematurity	115	24,4%
Perinatal asphyxia	71	15%
Neonatal respiratory distress	57	12,1%
Malformations	28	5,9%
Others	48	10,1%
TOTAL	471	100%

**Table 4:** Causes of mortality in children over 1 month

	1 month to 1 year	1 to 5 years	5 to 15 years
Pneumonia	19 (27,1%)	5 (11,1%)	–
Meningitis	7 (10%)	5 (11,1%)	–
Diarrheal disease	7 (10%)	–	–
Sepsis	–	–	5 (13,1%)
Malformations	13 (18,5%)	5 (11,1%)	–
Heart diseases	7 (10%)	3 (6,6%)	3 (7,8%)
Neoplasia	2 (2,8%)	5 (11,1%)	9 (23,6%)
Poisoning	3 (4,2%)	–	–
Traumatic causes	–	3 (6,6%)	3 (7,8%)
Scorpions stings	–	2 (4,4%)	–
Burns	–	3 (6,6%)	–
Unclear	12 (17,1%)	14 (31,1%)	18 (47,3%)
Total	70 (100%)	45 (100%)	38 (100%)

## Discussion

The national data on pediatric mortality is rarely available in Morocco [3, 4]. Our study is the first to lead an audit on pediatric mortality in a third level ward of the south region. During our study period, the mortality rate was at 3.63 per 1000. While comparing our results to similar available series from countries outside of Morocco, we found that our rate was way underneath that of the Bassey et al. [5] study with 27 per 1000, the Robison et al. [6] study (37.9 per 1000), the Ndu et al. [7] study (58 per 1000), the Joffiro et al. [8] study (41 per 1000) and the Santhanam study (122 per 1000) [9]. These disparities can be explained at least partially by the socio-economic level of each country. Morocco is still considered as developing country in spite of its rapid economy development. Moreover, our rate is higher that of the Zhu et al. [10] study with 0.5 per 1000. All these studies were conducted in developing countries. In developed countries, the death of children in pediatric emergency is rare and even has a tendency of dropping [11, 12]. Therefore our rate is largely superior to the 0.17 per 1000 admissions recorded in the Maniktala et al. study in the USA [13], and to the rate of 0.15 deaths per 1000 admissions recorded in the lopez et al. study in Spain [14].

Parents earning low income and residing in rural areas were the main risk factors of pediatric mortality around the world [15]. In our study, we had a high rate of children coming from disadvantaged areas. In fact, the mortality level was higher in low income households and within those living in rural areas. Financial and geographic inaccessibility on top of a parent’s low level of education may explain the high rate of child mortality in this group. Some studies found links between the mother’s social and economical factors and the mortality, morbidity and even the children’s congenital abnormalities. Understanding these associations may contribute to interventions that aim at better equity in the healthcare system [16, 17]. More thanone third of the deaths in our ward occurred in the winter season coinciding with the peak of bronchopulmonary infections. This observation is reported in other studies especially from Africa [5, 7]. Two-thirds of our patients died in the first 24 h compared to 32% for Jofiro et al. [8]. Our median of the period of stay in the PED was 12 h largely exceeding that of Zhu which was around 1,5 h [10]. Ideally, the period of stay of patients shouldn’t exceed 48 h. On the other hand, it was noted that the delay in the patients’ transfer to emergency units was associated to a higher mortality level. Therefore shortening the period of stay of patients in emergency units and avoiding any delay in their transfers to the intensive care unit may reduce the mortality rate [10, 18, 19]. Most of the deaths were recorded between 4 p.m. and 8 p.m. This could be explained by this period coincides with the departure of the medical team including the pediatric seniors and its reduction to the only pediatric resident on call in our ED.

In our study, neonatal mortality was dominant with a rate of 75.1%. The causes of this neonatal mortality were dominated by neonatal infections, prematurity and perinatal asphyxia. Although, this rate is high, it joins the data of other studies [9, 10, 20–22]. Neonatal mortality is a real tragedy of the public health especially in developing countries, even with its drop in all of the regions of the world. Between 1990 and 2017, the global rate of neonatal mortality dropped by 51% going from 36.6 deaths in 1990 to 18 per 1000 deaths in 2017 [22]. Although, a lot of substantial progress has been made to reduce the neonatal mortality in 1990, more efforts should be made to achieve the sustainable development goal by 2030 [22]. A global approach containing all of the mothers’ and the newborns’ aspects of health, with an improvement of the environmental and socioeconomic conditions of the mom, follow-ups of the pregnancy, medicalisation of the births and an improvement of the care given to the newborn seem necessary knowing that prematurity and perinatal asphyxia are still the main causes of mortality. On the other hand, increasing the number of adapted units and neonatology wards for a better approach of the healthcare provision seems to be a priority [20, 23].

Our study noted a high level of mortality (93.3%) in children under 5 years old in our hospital, joining other studies carried out in developing countries [7–9]. The main causes of this mortality were dominated by pneumonia and meningitis there by correlating with results from other countries [8, 10, 20]. With regards to children between 5 and 15 years old, we found that neoplasms were the main causes, similar to other studies made in China and Ethiopia [8, 10]. In our context, even though we have a pediatric oncology hospital, the lack of places for hospitalization and oncology-specific care explains this high mortality rate.

The infant sudden death syndrome was also reported as a cause of death in the Lopez et al. and Maniktala et al. studies with the following rates: 20.7 and 31% respectively [13, 14].

We couldnt explore more about this cause due to the criteria of our study that excluded children that were dead on arrival. In our context, the parents’ hesitation towards the practice of the autopsy make it hard to determine the causes of the infant’s sudden death syndrome. The same report was given by the Ntuli et al. study [20]. Zhu et al. reported a rate of 45.2% of children that were dead on arrival for whom no autopsy was done too [10]. On the other hand, other studies showed that more than the half of the deaths had one or multiple problems at the same time implying mainly severe asphyxia at birth, congenital defects, little weight at birth and prematurity [10, 13]. Bad nutrition and diarrhea were also comorbidities in Africa [8, 24]. This wasn’t found in our study. On another note, the indetermined mortality rate in our work (15.2%) was lower than the Zhu et al. study (20%) [10] and higher than the Ntuli et al. study (6%) [20].

Generally, our study will be helpful for emphasing the mortality of patients in PEDs in Morocco and maybe other developing countries. But it has some limitations. This study was done in a single PED and may not represent the rest of Morocco’s PED mortality rates. Therefore the results cannot be extrapolated to other departments. In addition, this study remains a retrospective study which made it difficult to give precise data on the death in some cases. There is also the problem of intricacy of more than one of concomitants factors or pathologies that made it complex to explore record data owing to the retrospective design of our study. We had excluded children dead on arrival and did not conduct autopsies, so all diagnosis remain clinical diagnosis. Thus will bias the interpretation of the results. On the other hand, it remains a simply descriptive study, so a large studies with multivariate analysis of the risk factors associated with the pediatric mortality is necessary in the differents emergency departments across our country.

## Conclusion

The analysis of the causes of death in the pediatric emergency units may help in the development of practical care and prevention. Our study keeps all its interest in being the first epidemiologic study of its type made in the biggest Pediatric Emergency Unit in the south of Morocco. It showed a relatively high mortality rate in children under 5 years old dominated by the neonatal mortality. Our data once again underline the crucial importance of prevention. This emphasizes the importance to increase the number of the neonatal intensive care units (NICU) and its impact to reduce neonatal mortality.

## Data Availability

The datasets used and/or analyzed during the current study are available from the corresponding author upon reasonable request.

## Abbreviation

PED: Pediatric emergency departement
